# Editorial

**Published:** 1913-03

**Authors:** 


### EDITORIAL

The Business Office of the *Journal-Record* is 23 West Alabama Street.

The Editorial Office is 1014-15 Atlanta National Bank Building.

Address all Business Communications to *Journal-Record of Medicine*, 23 West Alabama Street.

Make remittances payable to *The Journal-Record of Medicine.*

On matters pertaining to the Editorial and Original Communications, address Edgar G. Ballenger, M. D., Atlanta, Ga.

Reprints of Original Articles will be furnished at cost price. Requests foı the same should always be made in THE MANUSCRIPT.

We will present, postpaid, on request to each contributor of an original article, twenty (20) marked copies of *The Journal-Record of Medicine* containing such article.

### THE DIAGNOSTICE VALUE OF GONOCOCCIC VACCINE

The injection of 50 to 100 millions gonococci may cause a general reaction, similar to that produced by tuberculin, with pain in the infected areas such as joints, tendons, tubes, the prostate, and peritoneal or pleural infections; such results are often of value in making a diagnosis in obscure conditions.

The local diagnostic reactions have been advised; one is to scarify the skin and rub in a glycerin suspension of gonococci as in the Von Pirquet method. Bruck, Reiter and Irons have used this reaction. A positive reaction occurs in from twelve to twenty-four hours and appears as an area of hyperemia from 5 to 10 millimeters in diameter, or as a pauple. A control inoculation is made with a glycerin extract of the washings from the same number of uninoculated culture tubes. Neither this nor the following test is as reliable as the complement fixation reaction, which however, has the difficulty of being much more difficult to perform. The other method is to inject into the skin, not under it, two or three minins of a suspension of dead gonococci in a strength of 50 to 100 million per c.c. The needle should be sterilized by boiling in plain water so as to have it free from chemical irritants. A small wheal forms at the site of the injection. If the reaction is positive in about 24 hours a red purple, surrounded by a zone of lighter red, is formed two or three inches in diameter. At times the whole area may be elevated. It usually fades in two days, but occasionally may last for a week. In discussing this reaction London says:

"In cases in which the causative agent of an affection, such as synovitis, is known, the skin reaction may be negative. In seeking for an explanation in such a case it is probable, from present ideas of anaphylaxis, that the failure to react to foreign protein of a specific nature is as follows: In inactive cases—that is, in cases not progressive and not associated with septic phenomena—such as chills, sweats, fever, rapid pulse, emaciation, etc., the immunizing apparatus has not recently been stimulated to react against this specific irritant; in other words, it has not been sensitized. This may help to guide us in a choice between vaccine and serum treatment. If the defensive powers of the body do not produce antibodies to antagonize the specific proteins contained in specific germs, or their toxins, measures looking toward an active immunization by vaccines are apt to fail, and we must strive to obtain a passive immunization by means of protective sera, containing preformed the specific antibodies for the specific micro-organisms involved. This may help to account for the fact that widely different results are obtained in gonorrheal arthritis by the vaccine treatment. While some results have been brilliant, others have been disappointing; it must be borne in mind, however, that there are border-line cases that react to vaccination only after prolonged treatment, the first injections probably sensitizing the patient to the latter injections, thus ultimately causing the reaction and cure."

---

### REPORT OF COMMITTEE OF THE MEDICAL STAFF OF THE TABERNACLE INFIRMARY

To so ruthlessly take away from life and usefulness at a time when she seemed so much needed, a woman who had largely given her life to the service of others, is but another of the problems which the Divine Ruler alone can solve.

In taking Mrs. Kime away, we desire to express the profounditiy of our sympathy to Dr. Kime and his family in a loss which now appears almost irreparable and to express the hope that they may be comforted in their bereavement and supported by Divine direction.

We the members of the Medical Staff of the Tabernacle Infirmary recommend that these evidences of sympathy be published in the local papers, the Journal-Record of Medicine and a copy to be sent to the bereaved family.

We further recommend that they be spread upon the minutes of our meeting as an abiding record of our condolence.

Committee:

L. P. Stephens, M. D.,

E. C. Davis, M. D.,

T. C. Davison, M. D.

